# Using a Dance Mat to Assess Inhibitory Control of Foot in Young Children

**DOI:** 10.3389/fphys.2019.01302

**Published:** 2019-10-16

**Authors:** Nathália Petraconi, Giuliana Martinatti Giorjiani, Andressa Gouveia de Faria Saad, Terigi Augusto Scardovelli, Sérgio Gomes da Silva, Joana Bisol Balardin

**Affiliations:** ^1^Núcleo de Pesquisa Tecnológicas (NPT), Universidade de Mogi das Cruzes (UMC), Mogi das Cruzes, Brazil; ^2^Instituto do Cérebro (INCE), Hospital Israelita Albert Einstein, São Paulo, Brazil; ^3^Universidade Federal de São Paulo (UNIFESP), São Paulo, Brazil; ^4^Centro Universitário UNIFAMINAS, Muriaé, Brazil; ^5^Hospital do Câncer de Muriaé, Fundação Cristiano Varella (FCV), Muriaé, Brazil

**Keywords:** inhibitory control, dance mat, children, Go/No-go, foot, preschoolers

## Abstract

The development of motor response inhibition is critical during preschool years and has been associated with an improvement in gross motor coordination in this population. However, the assessment of inhibitory abilities in young children is challenging in terms of task selection and subject engagement, especially when investigating foot responses. Thus, the aim of this study was to describe a child-friendly Go/No-go paradigm to assess inhibitory control of foot based on a dance mat protocol. In this method, Go and No-go stimuli are modeled in the context of a fishing game, and behavioral responses are assessed by recording the latency to touch the mat and the accuracy of the touches. In this protocol article, we (1) describe the stages of the experimental set-up, (2) provide an illustrative data collection example in a sample of children aged 3–4 years, and (3) describe how to process the data generated. The utilization of the dance mat provides a feasible tool for researchers interested in studying the development of motor inhibitory control of foot in preschoolers. Potential applications of this protocol may include studies on developmental differences between hand and foot specialization, sports-related performance and neuroimaging.

## Introduction

Inhibitory control is important for numerous aspects of cognitive maturation during infancy. It is defined as: the ability to regulate focus on selective stimuli, suppressing irrelevant information (interference control or inhibitory control of attention); the ability to resist prepotent mental representations, such as unwanted memories and thoughts (cognitive inhibition); and the ability to prevent planned or ongoing movement from interfering in the performance of certain tasks or behaviors (response inhibition or motor response inhibition) (Carlson and Moses, 2001; Diamond, 2013; Brevers et al., 2018). Inhibitory ability enables children to solve complex problems, such as mathematical questions (e.g., switch the addition operation to subtraction) (Bull and Scerif, 2001; Crova et al., 2014), supports learning (e.g., suppressing distractive and attending to specific information) (Lee et al., 2015) and emotion (e.g., inhibiting negative expression in unpleasant situations) (Johnstone et al., 2005; Carlson and Wang, 2007). This ability is also important for the development of children’s motor activities. Impairment, such as developmental coordination disorder (DCD), and delays in motor skills have been associated with immature motor response inhibition, which may affect motor activities, including those that are automatic, such as walking (Ruddock et al., 2016; Bernardi et al., 2018). Studies suggest that children with an impairment to their motor skills present failures in cerebellar mechanisms responsible for motor control (Rigoli et al., 2012), resulting in a deficit of central control and sensory organization (Ruddock et al., 2016; Speedtsberg et al., 2017). Moreover, an abnormal connection between the frontal cortex (the predominant region for inhibitory ability) and the cerebellum is related to poor integrity of motor response inhibition (Rigoli et al., 2012; Bernardi et al., 2018; He et al., 2018). Children in these conditions may present poor postural control, smaller step length, slower velocity during the task and continuous imbalances, increasing the incidences of falling (Deconinck et al., 2010). Furthermore, this atypical activity may affect motor planning and execution which may slow the motor response or cancelation of ongoing action (Schachar et al., 2007; Speedtsberg et al., 2017). Motor inhibition abilities have also been positively correlated with motor prediction, which is characterized as a re-organization of movement according to environmental conditions (Ruddock et al., 2015, 2016). Problems in predictive control of movements are associated with disturbances in fine and gross motor skills and poor performance of executive function, including inhibitory response (Wilson et al., 2013; Adams et al., 2017). An evaluation protocol could also be used to identify disorders related to a weak inhibitory control, such as DCD and Attention Deficit and Hyperactivity Disorder (ADHD) (Berryessa, 2016). Therefore, there is a need for assessment procedures of motor inhibition abilities suitable for young children, particularly to investigate foot responses at behavioral and brain levels.

However, assessing motor inhibitory function in preschool children is challenging. For instance, common inhibitory tasks use abstract stimuli that can be difficult for young children to understand. Moreover, adult-like paradigms fail to engage the interest of young children. Therefore, an increasing number of developmental cognitive neuroscience studies have developed child-friendly versions of common executive function tasks (for example see Perlman et al., 2016). Among the adaptations made by these studies are the provision of a coherent story line and the use of engaging graphics. The development of tasks that are more child-centered is thus crucial for a valid and reliable evaluation of motor inhibition in the first stages of the lifespan.

Paradigms suitable for a child may need to consider that the first years of life are a critical period for inhibitory control processes. Studies suggest that inhibition may present developmental signals at about 12 months of life, however, at around 3 years of age children show important gains in inhibitory ability (Booth et al., 2003; Wiebe et al., 2011). The development of inhibition seems to be related with frontal lobe maturation which is also marked during infancy and continues to develop through adolescence and adulthood (Carlson and Moses, 2001; Luna et al., 2004). Furthermore, evidence indicates that the cerebellum and the frontal lobe have a parallel development, which is associated with improvement of motor and cognitive skills, including motor response inhibition, in first infancy (Diamond, 2000; Booth et al., 2003; Gilbert et al., 2011; Wu et al., 2017). Therefore, appropriate child tasks may offer evidence of the development of the inhibition ability in behavioral and neuroimaging studies.

The assessment of inhibitory control abilities has been widely carried out using Go/No-go tasks. The classical version of the Go/No-go task requires participants to make a motor response to one stimulus category (the Go condition), and to withhold the response to another class of stimulus (the No-go condition) (Liu et al., 2013). In the context of evaluating preschool children, Wiebe et al. (2012) adapted the Go/No-go task using a fishing game scenario. The authors instructed the children to catch the fish (pressing the button with their hand) and avoid catching the shark (not pressing the button). They found that children were capable of responding to the task, and also showed that with age, the young children become more strategic and responsible for their responses in inhibitory tasks as they progressively improved in accuracy and speed between conditions (Go and No-go) and ages (Wiebe et al., 2012). Also using the fishing game format, Howard and Okely (2015) examined differences between a standard button press version and a touchscreen version in a sample of preschoolers. However, no studies have yet proposed an adaptation to the Go/No-go task which examines foot responses during first infancy.

Foot structure is essential for regulating balance and locomotion and enables young children to develop motor and social skills (Price et al., 2018). Compared with the hand, the foot is less stimulated by the environment, which means that it develops later. As a result, investigating foot development may be a better indicator of maturational processes (e.g., myelinization and dendritization) and hemispheric specialization (limb lateral dominance) (Bushnell and Boudreau, 1993; Gabbard, 1993, 1996). Moreover, Tabu et al. (2012) reported that hand and foot responses activated the same brain areas in an inhibitory task with adults, suggesting that the foot could be an appropriate alternative limb for evaluating inhibitory control. Therefore, assessing foot responses in preschoolers could offer more precise indications of the development of the brain mechanism of inhibitory control than an assessment using hand responses.

The purpose of the current study was to describe a protocol with a child-friendly modified version of the Go/No-go task proposed by Wiebe et al. (2012) to assess motor inhibitory control of foot in young children using a dance mat. Here we describe the development of the paradigm and its implementation, as well as results of an illustrative assessment of a sample of 3–4-year-old preschoolers.

## Materials and Methods

### Equipment and Setup

**FIGURE 1:**
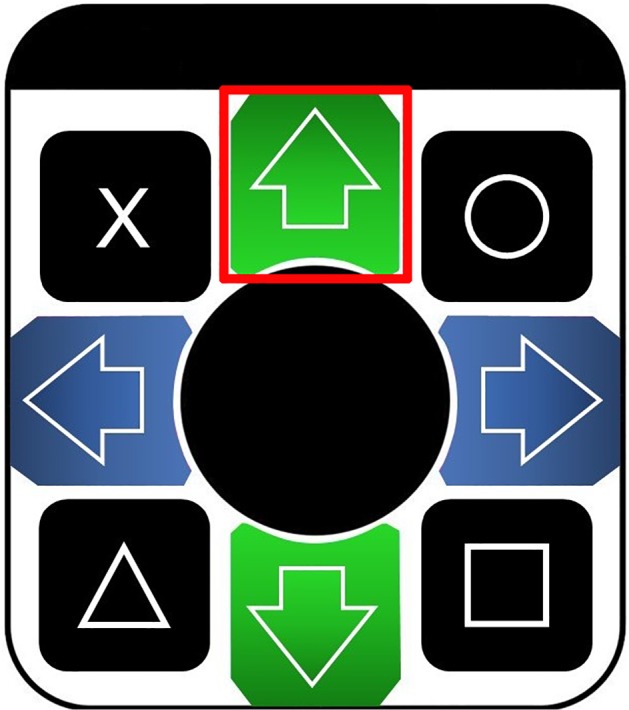
Dance mat example for use in Go/No-go task with foot protocol. The selected button in highlighted in red.

(1) Dance mat: to be used as a button-press response device for foot, suitable for preschooler populations (example, Dance Mat of DDR Game). The dance mat consists of equipment with the dimensions 36 × 32 × 1/4 inches and a 6-foot long cable (Figure 1). The dance mat may need a USB 2.0 adaptor and should have its configuration installed in the computer. The computer will recognize the mat as a peripheral joystick with buttons and axis. Select one of the buttons to be coded by the stimuli presentation software, preferably one that is easily accessible by the child. The task requires only one active button, so the remaining sensors should be covered to reduce potential distractors.
(2) Computer hardware and software to generate stimuli: stimuli can be presented via any option of hardware/software configuration that can smoothly display visual stimuli and record responses (with millisecond accuracy), for example DMDX^1^ or Presentation (Neurobehavioral Systems)^2^. Stimuli can be displayed on a computer screen or projected. Pictures should be big enough to be easily discriminated, for example 1280 × 720 pixels, and positioned in the center of the screen.

### Visual Stimuli

The fishing game protocol using a dance mat was programmed as a functional near-infrared spectroscopy (fNIRS) paradigm. The fish and shark stimuli times were based on the Wiebe et al. (2012) study. They found that children between 3 and 4 years of age were able to respond to 1500 ms stimuli using their hands. However, our protocol proposed a foot version of the task. Tabu et al. (2012) showed that feet respond more slowly than hands in adults. Wiebe et al. (2012) also verified that more than 2000 ms would be too long for children of this age, so we proposed 2000 ms of stimuli as appropriate for a foot response. The duration of the intervals during the task (interstimulus interval and resting block) were based on the attention level of the children and on the brain’s hemodynamic responses (Wiebe et al., 2012; Zamorano et al., 2014; Walsh et al., 2017; Herold et al., 2018). The whole experiment lasted around 5 min, which is the length of time recommended by Aslin et al. (2015). The number of stimuli (Go and No-go) presentations was established based on the studies by Wiebe et al. (2012) and Wilcox and Biondi (2015).

#### Stimuli Programming

**FIGURE 2:**
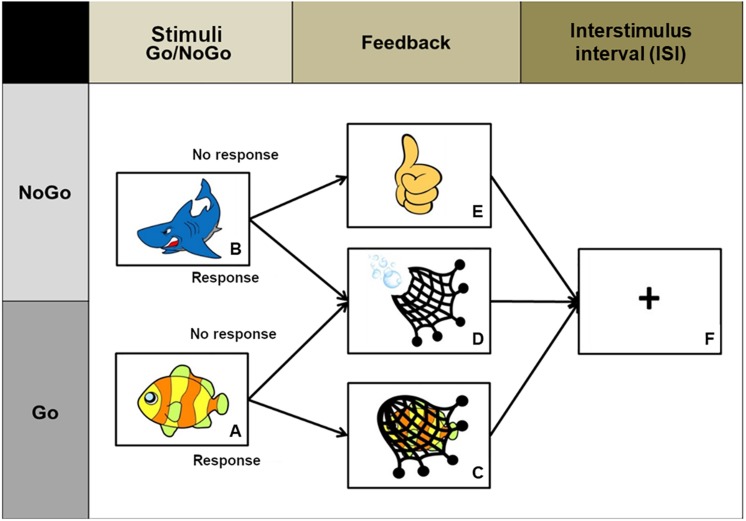
Schematic representation of Go and No-go trials, feedback for correct response and errors and the ISI image. **(A)** Go stimulus; **(B)** No-go stimulus; **(C)** feedback for correct accuracy; **(D)** feedback for misses and false alarms; **(E)** feedback for correct rejection; **(F)** Interstimulus interval (ISI).

**FIGURE 3:**
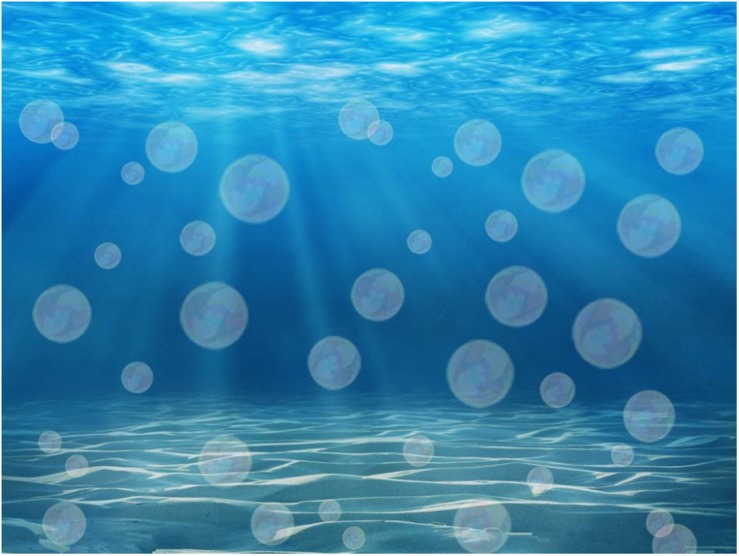
Static representation of the video interval between the Go and No-go blocks.

(1) Select a child-friendly drawn picture of a fish and a shark to comprise, respectively, the Go and No-go stimuli. Also select picture drawings for feedback stimuli (for example, a fishing net) (Figure 2).
(2) Design a paradigm for stimuli presentation. Stimuli are presented in a blocked fashion. In the Go block, the picture of the fish should be presented 7 times (duration 2000 ms). In the No-go block, also present the fish 7 times and randomly present the shark 3 times within the block (shark duration 2000 ms). Alternate the presentation of three Go and No-go blocks, interleaved with a 15 s-resting block (Figure 3). The presentation of task conditions is fixed and always starts with a Go block followed successively by a No-go block.
(3) Show different feedback screens (duration 1000 ms) in response to corrected responses to the fish (Figure 2C), misses to the fish (Figure 2D), corrected responses to the shark (Figure 2E) and false alarms to the shark (Figure 2D). Add a fixation cross after the feedback screen as an interstimulus interval (random duration 1000–2285 ms).
(4) Configure the fish and shark screens to record responses (i.e., touches to the dance mat).
(5) The duration of the task should be around 5 min.

### Procedure

Ensure that the study protocol is approved for use by the appropriate Human Subjects Committee. The protocol described here was approved by the Ethical Research Committee of the Universidade de Mogi das Cruzes, from Mogi das Cruzes, Brazil (approval number 2.626.590).

#### Task Description

(1) Obtain written informed consent from parents or legal guardians and the child’s assent.
(2) Tell children that they will take part in a fishing game. Position the child in the center of the mat, in a place without sensors, then explain the aims of the task: tell the children that they must catch the fish every time it appears on-screen by stepping on a particular button on the mat, the experimenter should show which button this is, and that they should not catch the shark, telling them “let the shark go home” to facilitate understanding of game’s goals. The instructor also tells them that the fish swim fast and that they should make sure not to let the fish escape. To differentiate the conditions (Go and No-go) use familiar and easily distinguishable images (e.g., fish and shark) for the preschool phase and present the goals in a transparent way, “catch the fish, but do not catch the sharks” (Wiebe et al., 2012). The experimenter should model the instructions for the child by stepping on the answer button on dance mat, and should stay with the child throughout the whole experiment, giving them voice feedback for correct answers and errors.
(3) Adjust the monitor location to the height of the children’s eyes, providing a target that facilitates their balance (Wittenberg et al., 2017). Instruct the children to keep standing in the center of the mat (i.e., a place without sensors). The child should perform a training session, based on a No-go block, to ensure task comprehension. Depending on the specificities of the study’s objectives, the child can use a predefined foot (right or left) to perform the task.

#### Outcome Measures

The computer hardware and software to generate the stimuli may be programmed to record outcome variables. In the foot version of Go/No-go tasks, measurements included Go accuracy (responses to the fish), false alarms (responses to the shark), correct rejections (no responses to the shark) and misses (no responses to the fish). The dance mat protocol was also programmed to record the reaction time of Go accuracy, related to the fish stimulus, and the reaction time of false alarms, related to the shark stimulus, i.e., the time that children took to respond to the specific stimuli after they appeared on the screen. The correct responses (Go accuracy and correct rejections), omission (misses) and commission (false alarms) errors may be computed singly and be compared in order to verify the difficulty level of the task. As reported by Wiebe et al. (2012), it is expected that children present around 75% Go accuracy and about 25% errors. It is also expected that the reaction time of Go accuracy is slower than reaction time of false alarms.

## Illustrative Data Collection

We evaluated a sample of 31 children (14 boys, 17 girls) from a public preschool in Mogi das Cruzes, São Paulo state, Brazil. The children were between 3 and 4 years old, with a mean of 3 years and 6 months, and had no history or evidence of neurological disorders. Written consent was obtained from all of the parents (or legal guardians), and verbal assent was obtained from all of the participants. The Affordances in the home environment for motor development (AHEMD) questionnaire was used to assess the influence of domestic environment on motor development (Rodrigues et al., 2005). To evaluate the level of motor development, we used the Test of gross motor development second edition (Ulrich, 2000) that assesses 12 motor abilities related to locomotion and object manipulation. The participants’ dominant foot was determined according to their performance on the kick-a-ball ability task from TGMD-2. In our sample, 9.7% were classified as left-foot dominant and 90.3% as right-foot dominant. Table 1 describes the demographic characteristics of the sample and the AHEMD and TGMD-2 results. Table 2 describes the results for the main outcome measures of the Go/No-go procedure. Prior to calculating the measures, trials in which RT were <300 ms were removed from analysis as they were considered too fast to be a valid response to the stimuli (Howard and Okely, 2015; Magnus et al., 2017). Regarding task compliance, all children completed the task and one child committed a great number of errors.

**TABLE 1:** Description of the sample according to the ranking in AHEMD and TGMD-2.

**Subjects**		**AHEMD (%)**			**TGMD-2 (%)**		
**Number**		**Low**	**Moderate**	**High**	**Low**	**Moderate**	**High**
Participants	31	22.60	70.95	6.45	22.60	64.50	12.90
							

**TABLE 2:** Descriptive data for the foot version of the Go/No-go task. Results are expressed as (mean ± standard deviation and median and min-max interval) percentage of Go accuracy, correct rejections, misses, false alarms, reaction time of Go accuracy and reaction time of false alarms of foot in the blocks Go and No-go.

	**Go Block**	**No-go Block**
Go Accuracy	86.64 ± 8.90% 85.71% (61.90–100)	84.49 ± 11.40% 85.71% (47.62–100)
Reaction Time of Go Accuracy	1099 ± 200.4 ms 1114.42 ms (627.75–1386.99)	1140 ± 230 ms 1183.23 ms (369.94–1440.43)
Correct Rejections	–	71.33 ± 25.46% 77.78% (0–100)
Misses	13.36 ± 8.90% 14.29% (0–38.10)	15.51 ± 11.40% 14.29% (0–52.38)
False Alarms	–	28.67 ± 25.46% 22.22% (0–100)
Reaction Time of False Alarms	–	884.2 ± 382.2 ms 869.8 ms (352.9–1549)

The proportion of correct Go responses was high for both Go and No-go blocks, suggesting the same level of performance in response selection (as demonstrated in Table 2). The task was also sensitive enough to prompt an average number of commission errors (i.e., false alarms - falsely pressing the button in No-go trials), which is commonly used as an interference measure to assess behavioral performance.

The results of this illustrative data collection suggest that the dance mat provides a feasible tool for researchers interested in studying the development of motor inhibitory control of foot in preschoolers. The format of the Go/No-go protocol presented here is particularly suitable for block-designed neuroimaging studies using fNIRS. The procedure is appropriate for use with very young children (3–4 years). Additional pilot testing may be required to adjust the rate of stimuli presentation when investigating samples with different age ranges and/or neuropsychiatric disorders.

## Ethics Statement

This study was carried out in accordance with the recommendations of Ethical Research Committee of the Universidade de Mogi das Cruzes with written informed consent from all subjects. All subject’s parents gave written informed consent in accordance with the Declaration of Helsinki. The protocol was approved by the same local ethical committee (approval number 2.626.590).

## Author Contributions

NP, SG, and JB conceived and designed the study and acquired, analyzed or interpreted the data. NP, SG, TS, and JB drafted the manuscript. GG, AS and TS contributed administrative, technical, or material support.

## Conflict of Interest

The authors declare that the research was conducted in the absence of any commercial or financial relationships that could be construed as a potential conflict of interest.

## Footnotes

1 http://www.u.arizona.edu/∼kforster/dmdx/dmdx.htm

2 http://www.neurobs.com
